# State Veterinary Associations

**Published:** 1883-10

**Authors:** 


State Veterinary Association.—We are reliably informed that a call is soon to be issued for a "State Veterinary Association." Mr. T. E. Daniels, proprietor of the *United States Veterinary Journal*, has been actively engaged during the last seven months in organizing State veterinary associations in various sections of the country. The movement began in Chicago and resulted in the formation of the Illinois State Veterinary Association. Associations have since been organized in Michigan, Wisconsin, Ohio, Indiana, Iowa and Pennsylvania. Considerable interest has been manifested and some good work done with the promise of greater results in the near future. The organization of the New York State Veterinarians into an association of a similar character will probably terminate his efforts in this direction for the present. Organizations like the one proposed are productive of much good. We need more unity of action, greater fraternity of feeling and much less jealousy and acrimony than the various cliques of the veterinary profession manifest at the present time. Our members are being yearly increased by the addition of excellent material in the shape of graduates from our flourising colleges and it is eminently proper that they should early become interested in the character of each other as well as the future advancement of the science of comparative medicine. We earnestly urge every graduated veterinarian to give his personal influence and presence to the support of the measure. The right kind of work in the beginning will insure success and make the veterinary profession of this State what it ought to be—a united, influential body of intelligent men.

